# Acceptability of computerized cognitive training and global cognitive stimulating‐based games delivered remotely: Results from a randomized controlled trial to address cancer and cancer‐related cognitive impairment in breast cancer survivors

**DOI:** 10.1002/cam4.5904

**Published:** 2023-04-11

**Authors:** Diane Von Ah, Adele Crouch, Susan Storey

**Affiliations:** ^1^ College of Nursing, The Ohio State University 1585 Neil Avenue Columbus Ohio 43210 USA; ^2^ NewCourtland Center for Transitions and Health, University of Pennsylvania School of Nursing Philadelphia Pennsylvania USA; ^3^ School of Nursing, Indiana University Indianapolis Indiana USA

**Keywords:** acceptability, BrainHQ, breast cancer survivors, cognitive training, crossword puzzles

## Abstract

**Background:**

Although the cancer‐related cognitive impairment (CRCI) is a common symptom incurred by the breast cancer survivors (BCS), more emphasis is needed in identifying acceptable interventions for survivors.

**Purpose:**

The purpose of this qualitative descriptive study was to examine the acceptability of cognitive programs by identifying the facilitators and barriers for implementing computerized cognitive training (BrainHQ) and computerized global cognitive stimulating‐based games (e.g., computerized word‐find, puzzles, etc.) comparator delivered remotely to improve CRCI from the perspective of BCS.

**Methods:**

BCS (*n* = 35) who enrolled and completed a randomized controlled trial of computerized cognitive training: 19 cognitive training (BrainHQ) and 16 global cognitive stimulating‐based games (crosswords, puzzles, etc.) were interviewed post‐training. Semi‐structured questions were used, recorded, and transcribed verbatim. Qualitative data were analyzed using standard content analytic procedures for each intervention.

**Results:**

Facilitators of training varied by intervention with cognitive training seen as challenging, engaging, and gave a sense of accomplishment whereas global stimulating games were seen as a way of taking mind off issues, enjoyable, and easy to navigate. Barriers of cognitive training included an awareness of failing whereas global stimulating games were deemed to be too repetitive. Both groups endorsed the convenience/flexibility of online training and common concerns of time constraints and fatigue to complete the training. Each group also provided recommendations for improvement.

**Conclusions:**

Cognitive training and global stimulating games were generally well received by BCS. Designing more support elements to promote engagement may be key to successful long‐term implementation.

## Graphical abstract

Cancer survivors often incur cancer‐related cognitive impairment (CRCI). Computerized cognitive training and cognitive stimulating‐based games have been used to improve CRCI. These methods have been shown to be well received and engaging; however more research is needed to engage patients in these programs for optimal results.

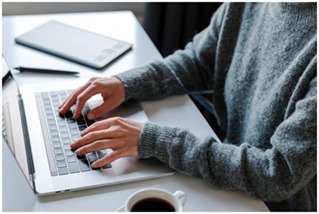

## 1 INTRODUCTION

Cognitive impairment after cancer and cancer treatment is a common problem for a substantial number of breast cancer survivors (BCS). Infact, up to 75% of cancer survivors report cognitive concerns^1^ which is highly significant given that there are over 3.8 million BCS in the United States alone.^2^ Cancer‐related cognitive impairment (CRCI) also has many downstream effects on everyday functioning,^3^ work‐related outcomes,^4^, ^5^, ^6^ and quality of life, including having a deleterious impact on social, psychological, and physical well‐being.^7^, ^8^, ^9^, ^10^ These effects are compounded by the fact there is a lack of evidence‐based clinical support for these BCS.

BCS are interested in treatment options for CRCI.^11^ The difficulty for providers is that there are no definitive evidenced‐based treatments available for cognitive concerns after treatment.^12^ Researchers have been exploring non‐pharmacological approaches to address CRCI, including exercise, mindfulness, and cognitive rehabilitative approaches (e.g., cognitive behavioral therapy, cognitive training, etc.); however, many of these studies have been underpowered to establish their effectiveness.^12^ The American Society of Clinical Oncology provides tips that include the use of mind‐stimulating activities or “brain‐strengthening” games such as crosswords or puzzles as potentially beneficial.^13^ Researchers have also noted that BCS utilize global stimulating‐based games or activities, such as crossword puzzles, word searches, Sudoku, etc.^10^, ^14^, ^15^, ^16^ to mitigate the effects of CRCI, whereas others have suggested that cognitive training programs may be useful.^1^, ^10^ In a web‐based survey, Lange et al. identified that BCS would use cognitive training (72%, *n* = 658) for CRCI^1^; however, the empirical evidence to support cognitive training for CRCI is limited.

Cognitive training programs focus on structured practice of cognitive tasks and generally include repetitive, problem‐orientated tasks that target specific cognitive domains in the effort to restore impaired skills.^17^ Cognitive training, which is based on the scientific principles of neuroplasticity or the brain's ability to reorganize and form new neural connections to accomplish tasks,^18^ may be a promising intervention for BCS. Neuroplasticity allows the neurons (nerve cells) in the brain to compensate for injury and to adjust their activities in response to new or changing situations, including cognitive training.^19^ Cognitive training is defined as “any intervention aimed at improving, maintaining or restoring mental function through the repeated and structured practice of tasks which pose an inherent problem or mental challenge”^20^ Evidence from animal and human studies provide promising evidence that cognitive training increases sensory stimulation, performance of cognitively challenging activities, promotes neuroplasticity, and improves cognitive outcomes.^18^, ^19^, ^21^, ^22^, ^23^, ^24^, ^25^ The procedural tasks required for cognitive training may lead to increasing brain activation and ultimately contribute to a sense of improved cognitive functioning and well‐being noted by the participant.^20^ These characteristics differentiate formal cognitive training from global stimulating‐based games. Global stimulating‐based games (e.g., crossword puzzles and games), are widely used by the public at large to support brain health and are strategy‐based games which generally do not provide the five core elements requisite of neuroplasticity including the principles of speed of processing, accuracy of processing, adaptivity, generalizability, and engagement. In comparison to the cognitive training programs, the global stimulating‐based games were not designed to leverage these principles (Table 1). These programs are both available via the computer for ease of use.

**TABLE 1:** Comparison of cognitive training versus global cognitive stimulating‐based games.

Element	Computerized cognitive training using BrainHQ	Computerized global cognitive stimulating‐based games
Visual stimuli	Intensive & repetitive with increasing visual stimuli	None
Complexity of stimuli	Progressively increased speed & processing of stimuli with increasing distractions	No progressive challenge, strategy‐based only
Cognitive demand	Tailored; training adjusted by computer to 85% of individual's threshold	Not tailored; not adjusted for individual threshold
Visual attention & memory	Directed attention with each exercise, precision	No directed attention from computerized intervention
Novelty	Individualized feedback and rewards built in to respond to the participant, game features to improve satisfaction	Feedback and rewards not provided, may incur some satisfaction on their own

Computerized cognitive training in older adults has shown promise in staving off the effects of advanced aging; however, those trials excluded older adults with cancer.^23^, ^26^ Research from studies using cognitive training to address CRCI in cancer survivors have also shown some promise.^6^, ^27^, ^28^, ^29^, ^30^ In our most recent double‐blind randomized controlled trial, we found that computerized cognitive training and computerized global stimulating‐based games were acceptable and satisfying on a brief quantitative scale for BCS with CRCI.^31^ However, this work and others has failed to thoroughly explore important contextual factors that may affect future implementation into practice. Glasgow et al. recommend that rapid learning research methods that assess and evaluate the feasibility of eHealth interventions should be conducted in the early research phase to determine factors that may affect future implementation.^32^ Qualitative research approaches are ideally suited to providing in‐depth contextualized accounts which in this case may serve to inform future research.^33^ Therefore, the purpose of this study was to examine the acceptability of cognitive training by identifying the facilitators, motivators, and barriers for implementing computerized cognitive training (BrainHQ) and global stimulating‐based games (e.g., computerized word‐find, puzzles, etc.) delivered remotely to improve CRCI from the perspective of BCS. Findings for this work provide information directly from the BCS in their own voice regarding the acceptability of completing training. Understanding key facilitators, motivators, and barriers to engaging in the cognitive training will provide insight for future interventional and translation research.

## 2 METHODS

This descriptive study used qualitative methods to assess acceptability of cognitive training and global stimulating‐based games to combat CRCI in BCS. The randomized controlled trial was completed at a Midwestern NCI‐designated cancer center. Upon completion of the randomized controlled trial,^31^ BCS in both groups who provided informed consent completed a 1:1 interview with the research assistant regarding their experiences. The study was approved by the Indiana University Institutional Review Board (Protocol #: 1703775084A008).

### 2.1 Sample

To be eligible for the original study, BCS were 21 years of age and older, had received chemotherapy as part of their adjuvant therapy for Stage 1–IIIA breast cancer, were ≥1‐year post‐adjuvant therapy (not including estrogen blocking therapy), and were disease free. For enrollment in the randomized controlled trial, all BCS had to report cognitive concerns (yes/no) and interest in receiving treatment for their cognitive concerns. In addition, all BCS in this study had fully completed the double‐masked randomized controlled trial testing computerized cognitive training against computerized global stimulating‐based games for improving CRCI.

BCS excluded from the original study had history of or current diagnosis that would directly impact cognitive function including stroke, traumatic brain injury, dementia, Alzheimer's disease or Parkinson's disease, history of or current other cancer (except for basal cell skin cancer), or history of invasive cancer treatments (brain surgery, history of cranial radiation or intrathecal therapy). In addition, BCS with current active major depression, substance abuse, history of bipolar disorder, psychosis, schizophrenia, or learning disability or participating in any other training were also excluded.

### 2.2 Procedure

The original parent study was a double‐blind randomized controlled trial designed to test two computerized cognitive training programs (refer to clinical trial #NCT05570604).^31^ BCS enrolled in the study were randomized to one of two groups–computerized cognitive training or computerized global stimulating‐based games. BCS were assigned to complete on average 4 h of training per week over a 10‐week period for a recommended total of 40 h. Cognitive training included the utilization of the commercially available BrainHQ program (Posit Science®), which was originally developed as part of the ACTIVE trial.^34^ This program systematically reduces the stimulus duration during a series of progressively more difficult information processing tasks presented via computer. The exercises automatically adjust to user performance to maintain an 85% correct rate. A variety of games were included to address the cognitive domains known to be most affected in BCS with CRCI, including memory, speed of processing, attention and working memory, and executive function.^35^ The games included exercises which required time‐order judgment, discrimination, spatial‐matching, instruction‐following, and narrative‐memory tasks.^36^ The group assigned to the global stimulating‐based games also completed online games via the computer. This group received global cognitive stimulating‐based games that offered a predetermined set of computerized crossword puzzles, word‐find, puzzles, and more. Primary outcomes included satisfaction with the intervention, effect size and reliable improvement of perceived cognitive function (cognitive abilities and cognitive concerns) and health outcomes, including work ability, health perception, (status and change) and quality of life. Secondary outcomes were performance on neuropsychological tests and plasma levels of brain neurotropic factor (BDNF). Data were collected at baseline and immediately post‐intervention. In addition, qualitative interviews were conducted post‐intervention for both groups to determine factors that may influence future implementation efforts.

### 2.3 Data collection

The interview had one over‐arching open‐ended question and three prompts to identify the acceptability of the training including: Can you tell me about your experience of being involved in this training? Prompts: (1) Facilitators and Motivators: Can you identify anything that motivated (influencing factors) you to do the training? Can you identify anything that facilitated (enabling factors) you to do the training? (2) Barriers: Can you identify anything that prevented you from doing the training? and (3) Based on your experience, do you have any recommendations for future cognitive programs? The final question allowed the BCS to add anything else that they felt would be beneficial to understanding their experience with the cognitive program assigned.

### 2.4 Data analysis

Using standard content analytic procedures, the team analyzed the data in several stages.^37^ Team members (DV and AC) read through the transcripts multiple times in order to fully understand how the BCS described their experience with the cognitive program assigned (BrainHQ® cognitive training or global stimulating‐based games). The primary investigator highlighted and extracted text units (e.g., phrases, sentences, or stories) that captured how the BCS described their experiences. The text units were coded with a word or phrase that captured their essence. The codes were verified by the research team members. The codes were then categorized and agreed upon through team discussion and consensus.

The primary investigator placed the codes into a case‐by‐topic table for data display.^38^ Cases were presented on the vertical axis and the categories were presented on horizontal axis. Codes were placed in appropriate cells. The codes in each column were summarized and a narrative description of the categories in each column was written by the primary investigator. The narratives were confirmed by the other team members through a review of the transcripts. Excerpts were also selected which best conveyed the essence of the narratives.

## 3 RESULTS

A total of 37 BCS completed the original randomized controlled trial. For this study, 35 of the 37 (95%) who enrolled and completed a randomized controlled trial of computerized cognitive training: 19 cognitive training (BrainHQ®) and 16 global stimulating‐based games (crosswords, puzzles, etc.) agreed to be interviewed post‐intervention. Two of the BCS who completed the global stimulating‐based games in the original study were not interviewed because: one incurred a stroke between study timepoints and data was eliminated from final analyses and one BCS was lost to follow‐up and did not complete the interview with the investigator.

Table 2 displays the characteristics of the sample stratified by intervention group. There were no significant differences in the demographics and medical information between the two groups. Overall, BCS participating in this study were on average 57.6 (SD = 8.2) years old, 6.2 (SD = 4.9) years post‐diagnosis, and the majority were married or living with a partner (65%). The sample included 57.6% BCS who identified as White and 42.4% as Black or African American. The majority had Stage I or Stage II disease (77.1%), had received surgery (88.6%), and radiation therapy (82.9%) as part of their adjuvant therapy. Post‐intervention interviews ranged from 15‐to 35 min in length, with the median length of the interviews recorded at 23 min.

**TABLE 2:** Characteristics of the sample (total *n* = 35).

		Intervention *n* = 19	Control *n* = 16	*p*‐value
Current age	Mean ± SD	56.3 ± 9.3	59.1 ± 6.8	0.622
Race	Black	8 (42.1%)	8 (50%)	0.774
	White	11 (57.9%)	8 (50%)	
Highest education	Mean ± SD	15.2 ± 1.9	16.5 ± 2.0	0.053
Marital status	Since/divorced/widowed	8 (42.1%)	4 (25.0%)	0.238
	Married/partnered	11 (57.9%)	12 (75.0%)	
Stage of breast cancer	Stage I	5 (26.3%)	4 (25.0%)	0.858
	Stage II	9 (47.4%)	9 (56.3%)	
	Stage III	4 (21.1%)	2 (12.5%)	
	Unsure	1 (5.3%)	1 (6.2%)	
Months since cancer diagnosis	Mean ± SD	63.7 ± 53.9	85.9 ± 64.6	0.275
Surgery (lumpectomy)	Yes	16 (84.2%)	15 (93.8%)	0.328
	No	3 (15.8%)	1 (6.2%)	
Radiation	Yes	17 (89.5%)	12 (75.0%)	0.275
	No	2 (10.5%)	4 (25.0%)	
Tamoxifen use	Yes	11 (57.9%)	5 (31.2%)	0.084
	No	8 (42.1%)	11 (68.8%)	
Aromatase inhibitor use	Yes	8 (42.1%)	9 (56.3%)	0.892
	No	11 (57.9%)	7 (43.7%)	

*Note*: Race: participants identified as Black or White only so other races not listed. Surgery: yes = had surgery; no = never had breast cancer surgery. Radiation: yes = had radiation; no = never had radiation therapy. Tamoxifen use: yes = indicates current and/or past use of tamoxifen; no = never had tamoxifen. Aromatase inhibitor use: yes = indicates current and/or past use of aromatase Inhibitor; no = never had aromatase inhibitor.

### 3.1 Qualitative findings

The in‐depth qualitative interviews were designed to understand facilitators (influencing factors) and motivators (enabling factors) as well as the barriers to completing the assigned cognitive program (e.g., BrainHQ® or global stimulating‐based games). The following summary provides acceptability information regarding each program as well as areas in which the two programs shared responses. Table 3 displays the excerpts of facilitators (influencing factors) and motivators (enabling factors) and corresponding themes noted for each intervention and for both programs combined. Table 4 provides the excerpts and corresponding themes for the barriers for each program and the programs combined. Table 5 summarizes the themes for the facilitators, motivators, barriers, and recommendations by BCS to enhance their experience with cognitive training program interventions.

**TABLE 3:** Facilitators and motivators for cognitive training and global cognitive stimulating‐based games.

Training program	Excerpts	Themes
Cognitive training specific (BrainHQ)	*#05*: *I liked challenging myself to do it*. *I enjoyed the games and the scenarios*. *I found them highly engaging*. *I would get kind of competitive about it and seeing if I could do better than I had the time before*. *#44*: *I really loved them* (*exercises*). *So*, *it was very easy for me to try to find time and try to learn as much as I could*.	Engaging
	*#13*: *I liked the variety of programs and the variety of being able to accomplish something and move on to something else…*. *I just liked there was always a variety*. *You weren't doing the same thing over and over and over again that was the best part about it*. *#16*: *I liked how there were different types of games*, *some verbal that would speak to you*, *those kind of things I really felt were helpful*.	Variety of games
	*#13*: *The exercises gave you a form of accomplishment that you could achieve something*. *#44*: *It gave me a real sense of mastery*, *and I felt good about doing something that I felt was helping my brain*. *#49*: *The things that I noticed in my brain*, *the changes*, *the concentration that I have in all areas…I can concentrate more and understand everything*.	Sense of accomplishment
Global cognitive stimulating‐based games	*#07*: *I looked forward to it because it took me away from everything for a while*. *#04*: *It kind of took my mind away playing that little maze game trying to beat the game*. *#08*: *All in all*, *I really enjoyed doing them because it's relaxing to a certain extent because you have to totally concentrate on what you're doing at the moment*. *So*, *I thought that was good*. *It's a very in a zone kind of thing*	Took my mind away
Both programs	*#03*: *Global stimulation–I liked being able to go online and do it when I want to*. *#02*: *Cognitive training–I like the computer idea when I heard it*, *I was like okay because of course again that could be something to work within your schedule*. *#05*: *Cognitive training–I did to like the flexibility just do it on my own schedule at home*.	Convenience
	*#41*: *My motivation was the potential for improvement in cognitive thinking*. *#14*: *I just want to get better*. *I know that challenging your brain is vital to doing that*. *So that was one of my motivation*.	Commitment to help myself

**TABLE 4:** Barriers for cognitive training and global cognitive stimulating‐based games.

Training program	Facilitator excerpts	Themes
Cognitive training (BrainHQ)	#06: *there were some things I just couldn't get*. *It was really eye‐opening*. *I was like oh*, *I'm really not quick with this*. #03: *It wasn't anything that was done it was just accepting the fact of where I was*. *It's not easy to realize that you can't remember things* #13: *It made me feel like*, *Gosh*, *there is something wrong with my brain*. *…*. *it showed me that I wasn't the person I used to be*. *I mean when you're used to being fairly accomplished*, *thought highly of*, *I mean I did a good job*. *I did a great job and then I can't follow three fish floating around*. *It's hard*. *Really? I used to do really well at these kind of tests*.	Awareness of failing
General cognitive stimulating‐based games	#27: *I would want to have access to a different*, *a wider set of exercises*. #22: *It became very boring*, *because I anticipated or thought that the games would change over a period of time*.	Repetition
Both	#15: Global stimulation–*My children*. *I just have too much going on*, *it's so hard now*. #02: Cognitive Training‐ *As I mentioned*, *I'm very busy so as far as being able to sit down for that amount of time on a computer… Timeframe demands were not conducive for my schedule at all*.	Time demands/constraints
	#04: Global stimulation*–And after I sat in front of it all day doing my work*, *I really didn't want to spend another hour doing the games*. *#*05: Cognitive training*–I also just kind of noticed like at different times in the morning when it was quiet*, *and I'd get up and do it I tended to do better*.	Fatigue

**TABLE 5:** Facilitators, barriers, and recommendations for cognitive training and global cognitive stimulating‐based games.

	Facilitators/motivators	Barriers	Recommendations
Cognitive training (BrainHQ)	Engaging Variety of games Sense of accomplishment	Awareness of failing	More instruction using the program when initiating the program
Global cognitive stimulating‐based games	Took my mind away Enjoyment	Repetition	More variety
Both	Convenience Commitment to help myself	Time demands/ constraints Fatigue	Reminder cues More follow‐up support

### 3.2 Facilitators and motivators

#### 3.2.1 Cognitive training

BCS identified three main themes including *Engaging*, *Variety*, and *Sense of Accomplishment* as facilitators and motivators for participating in the BrainHQ® cognitive training. BCS in the cognitive training program provided several unique and positive aspects regarding their training experience. BCS identified that they “truly enjoyed” the BrainHQ® cognitive training program. When probed as to why this program was acceptable, three main positive areas specific to this program were identified which motivated them to complete the program. The BCS described how the program made them feel. BCS identified that they *liked the challenge and found the games engaging* as well as the *variety of the games* offered by the program. Many of the BCS also expressed a *sense of accomplishment* in completing the program. For example, one 64‐year‐old BCS noted:

I liked the variety of programs and the variety of being able to accomplish something and move on to something else. [This] Gave you a form of accomplishment that you could achieve something. I just liked there was always a variety. You weren't doing the same thing over and over and over again that was the best part about it.

Another BCS who completed the training noted that:

I liked challenging myself to do it. I enjoyed the games and the scenarios. I found them highly engaging. I would get kind of competitive about it and seeing if I could do better than I had the time before.

Importantly, it was reported by the BCS in the cognitive training program that completing each session gave them a *sense of accomplishment* which in turn led to important feelings that they were improving their cognitive abilities. As stated by another BCS,

It gave me a real sense of mastery, and I felt good about doing something that I felt was helping my brain.

Table 3 provides more excerpts supporting the three main themes of *Engaging*, *Variety*, *and Sense of Accomplishment* for the cognitive training intervention.

### 3.3 Global stimulating‐based games

BCS who completed the global stimulating‐based program summarized the games as enjoyable because for many it provided a distraction and allowed the BCS an opportunity to “*Take Their Mind Away*” from everything.

One BCS stated,

The word search that they had on there, I liked that and the little maze. I liked that. That kind of took my mind away playing that little maze game, trying to beat the game.

Another BCS stated their feelings regarding the program as follows:

All in all, I really enjoyed doing them because it's relaxing to a certain extent because you have to totally concentrate on what you're doing at the moment. So, I thought that was good. It's a very in a zone kind of thing.

The BCS expressed that they felt that their time was well spent and enjoyed the opportunity to focus on the task in front of them.

### 3.4 Common facilitators and motivators for both groups

BCS provided responses that were categorized in two common themes that facilitated their use of both programs. The themes included the *Convenience* and their *Commitment to Help Myself*. Specifically, the BCS believed the convenience and flexibility of completing the program online was crucial to their participation and use of the cognitive programs. Many BCS discussed the flexibility of working with the program at “their own pace” being able to complete the program on their own time and without the added “stress” and burden of coming to the cancer center to participate which for many made it possible for them to use the program (Table 3 for excerpts for this theme). In addition, the BCS in both groups expressed that the opportunity to complete the program was something that they were doing for themselves to address their cognitive concerns. One BCS relayed this point as follows:

I did it because it's a study to help, to see if any of it helps…then as I got in there to do it, I realized that it was helping me, that some of them that I did were helping me with my memory and remembering things, and so that was a motivation.

Another BCS stated:

My motivation was the potential for improvement in cognitive thinking. I would like to have a clear thought and be able to express a clear thought.

The BCS, in this study, were clearly concerned about their cognitive abilities and were motivated to try one of these non‐pharmacological training programs in the hopes that it would be beneficial. They expressed that given their concerns regarding their cognitive function that it was important for them to seek out and find opportunities to improve and/or maintain their cognitive abilities. Table 3 provides more excerpts to support these themes.

### 3.5 Barriers

#### 3.5.1 Cognitive training

BCS in the BrainHQ® cognitive training group did express some barriers specifically related to the cognitive training program. The difficulty level, especially when initiating the program, made some uneasy and frustrated. Many expressed feelings of insecurity and worry regarding their performance especially when initiating the program. One BCS stated:

At first, it kind of made me feel like an idiot, but after I got where I learned how to …. It did improve.

However, many expressed an *Awareness of Failing*. Many BCS conveyed becoming more acutely aware of their cognitive changes or deficits. Several BCS expressed a level of surprise and concern over their performance. One BCS stated that, “*It was really eye‐opening*. *I was like oh*, *I'm really not quick with this”* and another noted that “*It's not easy to realize that you can't remember things*.*”* The BCS acknowledged that it was important to them that the cognitive training program was engaging and progressed in difficulty level, which they found helpful; however, they also expressed a greater sense or an “acknowledgement” that they were not performing as they would have expected of themselves based on past achievements.

### 3.6 Global stimulating‐based games

The global stimulating‐based games had a variety of optional strategy games available, but BCS uniquely expressed this program needed more choices or a larger number of games be provided overtime. One BCS specifically stated:

*I would want to have access to a different*, *a wider set of exercises”* and that at times, *“it became very boring*, *because I anticipated or thought that the games would change over a period of time*, *like after doing it for a week or so*, *maybe get a new set of games*, *or a new*, *just new games*.

Overall, the BCS would like to see more variety and less repetition with the global stimulating‐based games available.

### 3.7 Barriers to cognitive training and global simulation

In compiling the results, two main barriers were identified for both the cognitive training and the global stimulating‐based games. The barriers to program utilization by BCS included *Time demands or constraints* and *Fatigue*. Many of the BCS identified that other demands (e.g., work, family, etc.) were often barriers to training. They identified the need to schedule the training, blocking out their calendars from other interruptions, and making it part of their everyday routine as options to overcome these barriers.

Fatigue was also identified as a barrier to training. While it was clearly an advantage that the training was available online via the web and on computer, many BCS expressed that after working all day on a computer they felt drained and getting back on the computer when they were tired was difficult. Many BCS also discussed that their cognitive performance was not as strong when they were tired after a long day of working. Table 4 provides the excerpts that correspond with these themes.

### 3.8 Recommendations for the future of cognitive training programs

Table 5 provides an overall summary of the themes related to the facilitators, motivators, barriers, and recommendations for improvement provided by the BCS in this study. BCS provided important information to help promote adherence for both mind‐stimulating training programs. BCS in the cognitive training program suggested more structured orientation to the program would be beneficial. They felt that some of the program options were initially difficult and would have liked more guidance in navigating the program. BCS assigned to the global stimulation suggested more variety of global stimulating‐based games along with feedback which would provide opportunities to know if they were improving.

BCS from both groups discussed their preference in having more support. They recommended implementing reminder cues for completing the cognitive programs. The BCS thought that reminder messages would aid them in completing the program each week in a timely manner. In addition, they favored options that included more customer support services including opportunities for more interactions regarding their performance. One BCS expressed it best by suggesting:

The only thing that maybe would be nice is if …someone would follow up with you and see how you're doing.

BCS identified that this additional follow‐up support could serve as both reminders to complete the program as prescribed as well as provide motivation and reassurance regarding their level of performance. In addition, it was believed that adherence was best when they made the program “part of their everyday schedule.” Both groups were consistent in their message that the program needed to be delivered remotely. As reinforced by one BCS, who stated that the remote home delivery was essential as follows:

I think that the accessibility of having it in your home. If you've got that, for me that's ideal. I think it's harder to actually have it all done in person. I think part of that is kind of more of a stressor. You can sit here at home and do it in your pajamas and you don't have to get up and go and find a place to park and do that kind of stuff. Online would be best in my view.

## 4 DISCUSSION

CRCI is a prevalent, bothersome, and potentially debilitating symptom for BCS and there are few evidence‐based treatments. This symptom has been reported more frequently by BCS who are increasingly requesting treatment.^1^ Although, the American Society of Clinical Oncology recommendations promote mind‐stimulating activities the evidence for this work needs further investigation.

Previous randomized controlled trials have suggested that cognitive training programs are acceptable.^31^, ^39^, ^40^ However, these studies failed to fully assess the facilitators, motivators, barriers, and recommendations for the pragmatic implementation of cognitive training programs. This secondary study was one the first to fully examine the acceptability of both specific cognitive training (BrainHQ®) and global cognitive stimulating‐based games (e.g., crossword, word finds, etc.) which may be used to address CRCI in BCS. Findings from this work provide insight from the perspective or experience of BCS themselves that will aid in implementing larger clinical trials and translational research as well as improve our understanding of implementing technology supported health behavior change.

Based on our findings and similar to other technologically enhanced health behavior programs, computerized cognitive training programs need to promote engagement. BCS in our study identified the need for more support and/or feedback. Researchers have identified that feedback is essential for user engagement, especially for promoting technology supported health behavior change, in this case the adoption of “exercising” or completing “mind‐stimulating” cognitive training.^41^ Specifically, the model developed by Cole‐Lewis et al. proposes that for optimal engagement that (1) the user interactions must promote or encourage use (e.g., rewards, social interactions) and make the experience appealing and (2) the health behavior intervention and its components must also be relevant to the individual.^41^ First, similar to this model, the BCS in this study, relayed that their experience was most favorable when they found the program was challenging, provided variety, and either gave them a sense of accomplishment or took their mind away to focus on the game‐related task. In addition, the ability to access the program from home promoted access and was seen as crucial to both cognitive training groups. This availability and flexibility were described as most appealing to the BCS and reduced the costs and stress associated with having to travel to a training facility. Second, the model identifies that engagement is higher when the health promoting intervention is highly relevant.^41^ The BCS in this study had already self‐identified as having cognitive concerns and seeking interventional options. The additional individual interviews highlighted that the BCS were committed to improving themselves. This commitment is instrumental in fueling the interest in engaging in the programs that were assigned and thus, future intervention trials should assess or gauge individual perceived need for the program. And finally, the barriers to the implementation of both programs mostly centered around aspects that hinder the user‐friendliness and appeal of the program. The cognitive training program which advances in difficulty level was noted as a positive but may also highlight performance concerns. Recommendations from this group of BCS included the need for more training and feedback regarding performance overtime. The global stimulating‐based games group identified the need for more variety of the games within the program. These changes are easily addressable and may promote adherence.

Recommendations from the BCS themselves, revealed that aids such as reminder cues would be instrumental to incorporating into this in their daily routine. In addition, implementing an individual training plan in which the cognitive training or global stimulating‐based games are preplanned will ensure that the training is a priority and may reduce the fatigue noted be those BCS who waited until the end of the day to complete their training. Similarly, Bail et al. noted that those BCS with the poorest adherence to the cognitive training program expressed being too tired or exhausted which ultimately, made focusing on the cognitive training program difficult.^42^ Thus, designing a plan that incorporates the cognitive training time at optimal times within each individuals' daily routine may promote training completion and engagement.

Finally, BCS from both groups identified more feedback or interaction from the program would be useful. Lampit et al. noted that individual home‐based cognitive training programs were not as efficacious as in‐person group‐based training; but this may have been because the web‐based programs failed to have individual support and feedback mechanisms.^43^ Thus, online computerized training programs delivered remotely must not only focus on technology support but should consider providing customer‐friendly support interactions to aid and coach BCS in completing the cognitive training. This interaction may help support optimal use of the program and provide feedback regarding performance. As identified by Cole‐Lewis et al. technology enhanced programs that provide encouraging support are appealing and promote usage and ultimately, engagement.^41^ Thus, upgraded support and coaching systems built‐within the program may provide optimal results.

### 4.1 Limitations

Several strengths and limitations of the current research must be considered in the interpretation of the findings. In terms of strengths, the novelty of this research must be noted, as it constitutes the first study of its kind to purposefully investigate the perspectives of BCS regarding facilitators and barriers to a cognitive program. In terms of limitations, to reduce subject burden of traveling to the center another time, the interviews were all conducted remotely. Conducting the interviews over the phone eliminated the ability to obtain non‐verbal communication or indicators that would have cued the investigator to probe more deeply than face‐to‐face interviews for this reason.^44^ We were also limited by the inability to interview those BCS who due to attrition or closing the study early due to the pandemic did not complete the original study and who may have had different experiences.

## 5 CONCLUSION

Cognitive training and global stimulating‐based games were overall well received. Interviews suggest that cognitive training, which advances in difficulty and provides more variety, is engaging. More research which facilitates engagement and reduces the barriers to web‐based computerized cognitive training interventions are needed to address CRCI after cancer and cancer treatment.

## AUTHOR CONTRIBUTIONS

**Diane Von Ah:** Conceptualization (lead); data curation (lead); formal analysis (lead); funding acquisition (lead); investigation (equal); methodology (lead); supervision (lead); validation (equal); visualization (equal); writing – original draft (lead); writing – review and editing (lead). **Adele Crouch:** Data curation (equal); formal analysis (equal); project administration (equal); writing – review and editing (equal). **Susan Storey:** Formal analysis (equal); writing – original draft (equal); writing – review and editing (equal).

## Data Availability

The datasets generated during and/or analyzed during the current study are available from the corresponding author on reasonable request.
